# Genetic drift of human coronavirus OC43 spike gene during adaptive evolution

**DOI:** 10.1038/srep11451

**Published:** 2015-06-22

**Authors:** Lili Ren, Yue Zhang, Jianguo Li, Yan Xiao, Jing Zhang, Ying Wang, Lan Chen, Gláucia Paranhos-Baccalà, Jianwei Wang

**Affiliations:** 1 MOH Key Laboratory of Systems Biology of Pathogens and Christophe Mérieux Laboratory, IPB, CAMS-Fondation Mérieux, Institute of Pathogen Biology (IPB), Chinese Academy of Medical Sciences (CAMS) & Peking Union Medical College, P. R. China, Beijing 100730, P. R. China; 2 Collaborative Innovation Center for Diagnosis and Treatment of Infectious Diseases, Hangzhou 310003, P.R. China; 3 Fondation Mérieux, Lyon, 69007, France

## Abstract

Coronaviruses (CoVs) continuously threaten human health. However, to date, the evolutionary mechanisms that govern CoV strain persistence in human populations have not been fully understood. In this study, we characterized the evolution of the major antigen-spike (S) gene in the most prevalent human coronavirus (HCoV) OC43 using phylogenetic and phylodynamic analysis. Among the five known HCoV-OC43 genotypes (A to E), higher substitution rates and *dN/dS* values as well as more positive selection sites were detected in the S gene of genotype D, corresponding to the most dominant HCoV epidemic in recent years. Further analysis showed that the majority of substitutions were located in the S1 subunit. Among them, seven positive selection sites were chronologically traced in the temporal evolution routes of genotype D, and six were located around the critical sugar binding region in the N-terminal domain (NTD) of S protein, an important sugar binding domain of CoV. These findings suggest that the genetic drift of the S gene may play an important role in genotype persistence in human populations, providing insights into the mechanisms of HCoV-OC43 adaptive evolution.

Coronaviruses (CoVs) are widely found in humans as well as in mammalian and avian species, causing asymptomatic infections or respiratory tract disorders, and gastroenteritis of varying severity^1^. CoVs belong to the genus Coronavirus of family *Coronaviridae*, and are classified into four genera, *Alphacoronavirus* (α-CoV), *Betacoronavirus* (ß-CoV), *Gammacoronavirus*, and *Deltacoronavirus* based on phylogenetics and serology^1^. To date, α-CoV [including human CoV (HCoV)-229E and NL63] and ß-CoV [including HCoV-OC43, HKU1, Severe Acute Respiratory Syndrome CoV (SARS-CoV) and Middle East Respiratory Syndrome CoV (MERS-CoV)] are known to infect human beings^1^.

Interspecies transmission is a common phenomenon for CoVs that may be responsible for generation of new CoV epidemics during viral evolution^2^,^3^,^4^,^5^. For instance, the feline CoV (FCoV) type II, a member of α-CoV, might be generated by a double recombination between FCoV type I and canine CoV^4^. Interestingly, porcine hemagglutinating encephalomyelitis virus, bovine CoV (BCoV) and HCoV-OC43 of ß-CoV are thought to have evolved from the same common ancestor^5^. During evolution, high frequencies of homologous RNA recombination and gene mutations are considered the main forces that push CoVs to adapt to specific hosts. Such events can lead to emergence of new strains or genotypes within a certain species, and even to new species, causing epidemic or zoonotic outbreaks that continuously threaten human health^2^,^3^. This phenomenon is exemplified by the recent emergence of SARS-CoV and MERS-CoV^6^,^7^. However, the detailed evolutionary mechanism of interspecies transmission and the persistence of CoVs in specific hosts have yet to be fully elucidated.

CoVs have a positive-sense, single-stranded RNA genome, with a length of ~27–31 Kbs^1^. The spike (S) protein of CoVs protruding on the surface of virions is the major antigenic protein for inducing neutralizing antibodies. However, it is in turn under the highest selection pressure among the viral proteins^1^,^8^. S proteins are often cleaved into S1 and S2 subunits to achieve receptor binding and membrane fusion, respectively^1^. The S1 subunit is composed of two distinct domains, the N-terminal domain (NTD) and the C-terminal domain (CTD), which play important roles in receptor binding^1^. The NTD is responsible for sugar receptor binding in some CoVs, such as BCoV and HCoV-OC43, or for protein receptor binding in murine hepatitis virus^1^,^9^,^10^,^11^. The CTD functions as the protein receptor binding domains (RBD) for most of the CoVs^1^. The S1 subunits of all CoV genera have similar topological structure, preserved sugar-binding functions, but different receptors-binding functions, which suggest that subtly adaptive mutations occur in functional domains during evolution of CoVs^12^,^13^. Similar adaptive amino acids mutations around the receptor-binding region have also been found in norovirus, contributing to its epidemic in humans^14^. Investigation on the evolutionary insights of the S gene, particularly the functional domains, is imperative for understanding the evolution of CoVs and for tracing spillover events and ecological niches.

HCoV-OC43 is the most prevalent CoV in humans and the relatively abundant number of clinical cases and corresponding epidemiological data make it a good model for HCoV adaption evolution^1^,^5^,^9^,^15^,^16^,^17^. Although five genotypes (A to E) have been identified, genotype D has been the dominant OC43 genotype from 2004 to 2012^15^,^17^. Previous studies by our group and others have demonstrated that recombination contributes to the generation of new OC43 genotypes^15^,^17^, but little is known about how HCoV-OC43 genotypes persist in human populations. It is assumed that the continuous adaption of viral antigenic gene is required for the persistence of OC43 genotypes^18^. However, this hypothesis has not been carefully examined by precise evolutionary pattern analysis. In the present study, we characterized HCoV-OC43 evolution based on the phylodynamic and phylogenetic analysis of full-length S genes to provide insights into its transmission and the adaption.

## Results

### Relative effective population sizes of OC43 genotypes

**Figure 1:**
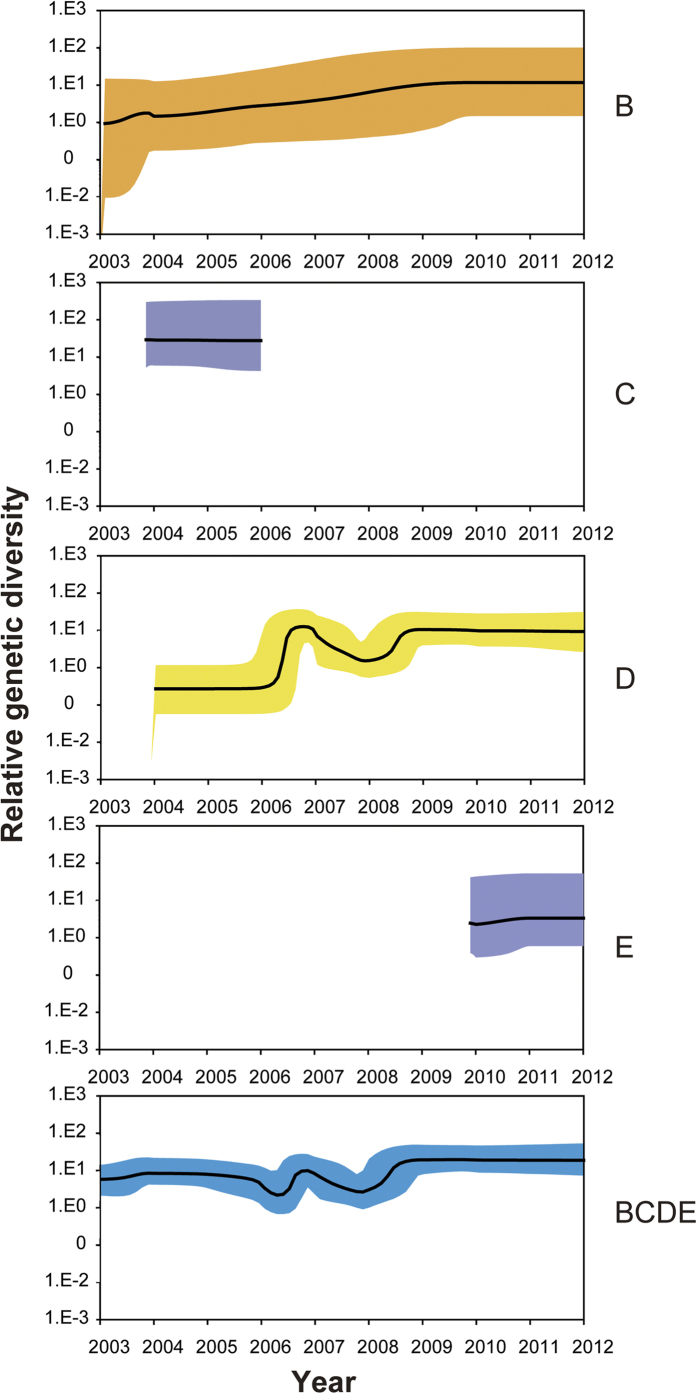
Relative genetic diversity dynamics of OC43 and each genotype. Population size was determined using sequences of 96 OC43 S genes obtained from 2003 to 2012. The median estimates (g) are represented by the black lines and 95% high posterior densities are shown in the color regions.

To verify the epidemic history of OC43 genotypes over the study period, the relative effective viral population size over time was inferred by analyzing the genetic diversity of the S gene using the Bayesian skyline model. Only the strains obtained from clinical samples containing the full length S, RdRp, and N genes retrieved from PubMed (http://www.ncbi.nlm.nih.gov) and GenBank (http://www.ncbi.nlm.nih.gov) for the years 2003 to 2012 were used (see Supplementary Table S1 on line). A varying population size profile was observed (Fig. 1). The overall plot of OC43 (including genotypes B, C, D and E) showed two large bottlenecks. The first was found between 2006 and 2007, showing a decrease in relative genetic diversity in 2006, followed by an increase in 2007. The second was found between 2008 and 2009, with a transient decrease before 2008, followed by an increase in relative genetic diversity coinciding with a temporal epidemic in population size. Genotype B exhibited a transient increase in 2004, then followed a steady but slowly increased relative genetic diversity, corresponding to global increases in its detection^17^. Genotype D showed a decreased genetic diversity before 2007, then an increase after 2009, consistent with its dominant epidemic from 2007 to 2012^17^. Genotype C and E both exhibited a short and steady relative genetic diversity during the study period. The history of the relative genetic diversity obtained here corresponds to global epidemic data, indicating that the relative genetic diversity measured by the S gene generally reflects the HCoV-OC43 dynamic in population size. The relative genetic diversity of genotype D corresponded to the two bottlenecks of OC43, indicating that genotype D accounts for much of the genetic diversity of OC43 and is the predominant genotype.

### Evolutionary rate of OC43 genotypes

To explore why genotype D became dominant after 2007, we calculated the evolutionary rate of OC43 genotypes. Using the constant population size under a relaxed-clock method, the mean evolutionary rate of the S gene was estimated to be 8.48 × 10^−4^ substitutions/site/year for OC43, consistent with previous reports^8^. For genotypes B, C, D and E, the mean evolutionary rate of S gene was 9.85, 4.85, 8.83 and 6.01 × 10^−4^ substitutions/site/year, respectively (Table 1). Similar results were obtained using the exponential growth model (Table 1). The highest evolutionary rates were observed in genotype B and D, suggesting that the two genotypes evolved faster than others.

### Natural selection of the S gene

To determine whether positive selection took place during the evolution of OC43, the *dN*/*dS* values and positive selection amino acids (aa) were calculated. The mean *dN/dS* ratio was observed in genotype D (0.31), followed by genotypes B (0.29), C (0.20) and E (0.15) (Table 2). Calculations for positive selection sites with a probability (Pr) of > 0.5 in the S gene identified 25 residues in genotype D, six sites in genotype B, one in genotype C, but none in genotype E (Table 2). Seven positive selection sites with Pr of > 0.9 were identified in genotype D. Further analysis showed that 12 of 16 positive selection sites of genotype D and 3 of 5 genotype B in S1 were located at the NTD (aa 15–312, reference strain 5240/07 KF572844), while four positive selection sites in genotype D and one site in genotype B were found in the predicted RBD (aa 339-549, reference strain 5240/07 KF572844)^15^.

**Figure 2:**
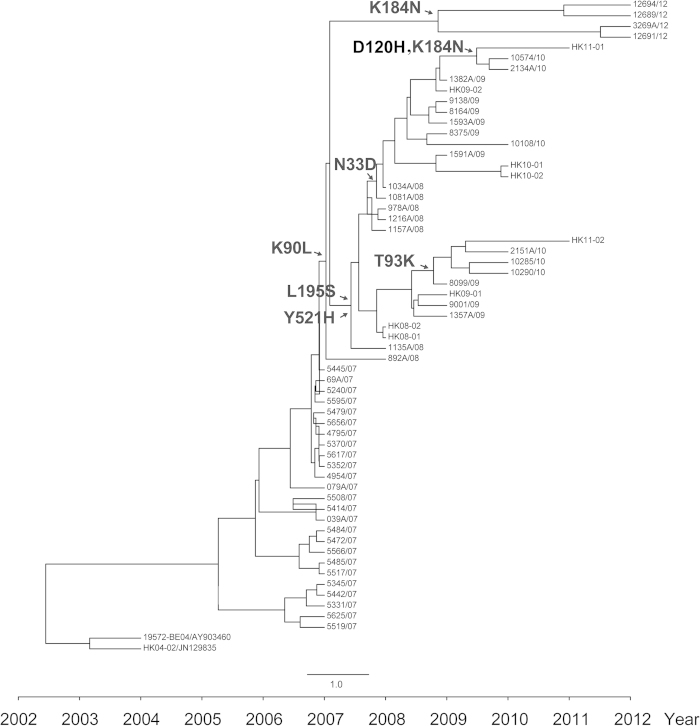
Molecular clock analysis of OC43 genotype D. The complete S gene sequences sampled from 2007 to 2012 were used to reconstruct the phylogeny. The calculated positive selection sites in each node are drawn on the MCC tree.

The relatively long epidemic time and more available sequences of genotype D allowed us to analyze in detail the nonsynonymous changes throughout the evolutionary history of OC43. The ancestral sequences were reconstructed and the occurrence time of each nonsynonymous substitution was estimated in the temporal evolution routes using the maximum clade credibility (MCC) tree. The residues at positions 33, 90, 93, 120, 184, 195, and 521 were positive selection sites, with six (33, 90, 93, 120, 184 and 195) located in NTD, and one (521) in the predicted RBD. All but one strain (5445/07) identified after 2007 contained the mutation of Y521H. All the strains identified after 2008 contained the mutation K90L. The mutations L195S and Y521H were found in strains identified between 2008 and 2011, but not in those of 2012. Other mutations, including N33D, T93K, D120H and K184N, were found in several strains. The chronologically traced positive sites might be associated with the ladder-like MCC phylogeny of the virus, indicating the key role of aa substitutions in the population dynamics of the virus (Fig. 2).

To confirm whether aa substitutions exist in NTD of other genotypes over time, the aa sequences of S from the 12 genotype B and 17 genotype C strains were also aligned. Eleven aa site mutations were observed in genotype B relating to the strain’s isolated over time, and seven sites were located in NTD (see Supplementary Fig. S1 online). No aa mutations were found in genotype C over time.

## Discussion

Our analysis on the global epidemic of OC43 in recent years showed the temporal transition of genotypes^17^. It is striking that the evolution of genotype D of OC43 is epochal among the four epidemic genotypes. First identified in 2004, it was generated by recombination^15^; after a stasis period in 2005 and 2006, a dominant epidemic was found over a longer timescale. As no new recombinant events were detected in the subsequently identified genotype D strains^17^, the mutations of the S gene−the major antigenic gene, likely plays an important role in driving viral epidemics^18^.

It is interesting that the positive selection sites calculated in S gene from different isolates over time are corresponding to the genetic variability of genotypes in this study. Genotypes B and D, which have high genetic variability contained more selections sites than that of C and E genotypes. The possible link between the positive selection sites and the genetic variability of genotypes should be evaluated in the future studies. The evaluation of relative genetic diversity, substitution rate, *dN/dS* value, and positive selection sites further showed that genotype D had a significant influence on the relative genetic diversity of OC43 and that its S gene evolved towards heterogeneity. More positive selection sites were also found in the S1 subunit of genotype D. Amino acid substitutions in the surface proteins are considered an important adaption strategy for the persistence of a virus to evade host immune pressure^8^,^18^. Although the neutralizing epitopes have not been identified, the substitutions identified in the S1 subunit of OC43 in this study are predicted to be in the antigenicity region (data not shown), which may allow viruses to escape host neutralizing antibodies^19^,^20^,^21^,^22^. Collectively, these findings suggest that genetic drift may play an important role in maintaining the spread of genotype D in the human population. Whether the genetic variations affect the related antigenic phenotype will need to be confirmed by antigen analysis using S genes with such mutants.

Most of the positive selection sites in S1 subunit are mapped in NTD. Molecular clock tree of genotype D also showed that 6/7 of the predicted substitution were located in NTD. The aa substitutions in NTD seem to be a common evolutionary strategy for CoVs, as the high variability in NTD has also been observed in BCoV and HCoV-NL63^21^,^22^. However, this observation warrants further investigation.

BCoV shares a high nucleotide and antigenic similarity with OC43^23^. The conserved sugar-binding sites identified in BCoV_NTD were also conserved in that of OC43 (see Supplementary Fig. S2 online), indicating the conservation of the core motif in NTD during the species-cross transmission and evolution in human hosts. It is interesting to point out that the evolutionary dynamic pattern of the conserved core and variable outer-region in NTD is similar to that of RBD observed in ß-CoV, suggesting that these functional domains retain some of the ancient records during viral evolution^12^.

The sugar moieties near the CoV receptor are considered critical co-factors to CoV infection. Antigenic analysis has predicted that there are some epitopes in this domain. Whether the aa mutations around the functional region of NTD are relevant to subtle remodeling of the binding process or antigenic evolution, like that observed in norovirus and influenza virus need to be investigated further^20^,^24^,^25^.

The RBD of OC43 has been predicted^15^. We found that the positive selection sites in RBD are less than those present in NTD. Notably, a Y521H substitution was found in the genotype D strains identified from 2008 to 2011. The significance of this mutation is unclear. It has been reported that a single aa substitution in RBD can cause marked antigenic differences and enable the virus to escape host immunity in influenza virus and norovirus^24^,^25^. Whether this single aa mutation can influence the host receptor binding activity needs to be investigated further. It is interesting that after 2012, the genotype D strains contained less mutations than those identified before 2012. The impact of these changes on the viral prevalence needs continuous surveillance of the OC43 genotypes in the future.

In summary, we report a model for the persistence of OC43 genotypes in human populations based on the first intensive evolutionary analysis of the S gene. We infer that the genetic drift of the S gene is likely to be one of the mechanisms of the adaptation evolution of HCoV-OC43. These findings provide insights into the evolution of CoVs and may have implications in the surveillance of HCoV infections.

## Methods

### Sequences and phylogenetic analysis

The full length sequences of OC43 S gene available in GenBank (http://www.ncbi.nlm.nih.gov) were retrieved on 30 May 2013, and analyzed together with the sequences identified previously by our group^17^. A total of 96 full-length S gene sequences obtained from clinical samples were used for analysis. The sequences were aligned using Clustal W program implement in MEGA 5.1^26^. The genotypes of these sequences were determined as reported^15^,^17^, including 12 genotype B, 18 genotype C, five genotype E and 61 genotype D. The background information of the sequences including accession numbers, collection dates, isolation areas, and genotypes can be found as Supplementary Table S1 online.

### Evolutionary analysis

The demographic histories and evolution rates of different OC43 genotypes were determined based on S gene sequence data by the Bayesian Markov Chain Monte Carlo (Bayesian MCMC) method implemented in BEAST (v1.8.1), using a relaxed molecular clock (uncorrelated lognormal-distributed model)^27^. The best substitution models were selected using Modeltest (version3.7) according to Akaike information criterion (AIC)^28^. The constant size and exponential growth tree models were used for the inference. Each Bayesian MCMC analysis was run for 100 million states and sampled every 2,000 states. Posterior probabilities were calculated using Tracer (version 1.5). The trees were annotated by the Tree Annotator program implemented in the BEAST package and the MCC tree was visualized using Figtree software (version 1.3.1). Bayesian skyline plots for OC43 genotypes were estimated to depict the relative viral genetic diversity over time.

### Positive selection analysis

To infer the positive selection sites of S gene at aa level, the deduced aa sequence entropy was determined using BioEdit (version 7.2.5)^29^. The ratios of nonsynonymous (*dN*) to synonymous (*dS*) substitution were estimated to evaluate the selection pressures on the OC43 S genes, using the codon-based phylogenetic method in CODEML (distributed in PAML, version 4)^30^. Posterior probabilities of the inferred positive selection sites were calculated using the Bayes empirical Bayes (BEB) approach which accounts for sampling errors^31^. The chronological evolution of *dN* changes throughout the evolutionary history of OC43 genotype D was traced using HyPhy software (version 2.2) and *dN* substitution was estimated using the maximum clade credibility (MCC) tree generated from Bayesian MCMC molecular clock analysis^27^,^32^. The aa sites were positioned according to the HCoV-OC43_D 5240/07 (KF572844).

## Additional Information

**How to cite this article**: Ren, L. *et al.* Genetic drift of human coronavirus OC43 spike gene during adaptive evolution. *Sci. Rep.* **5**, 11451; doi: 10.1038/srep11451 (2015).

## Supplementary Material

Supplementary Information

## Figures and Tables

**Table 1:** Evolution rate of the OC43 S gene in different genotypes.

Genotype	Substitution rate (CR)^a^	
	Constant size	Exponential growth
All	8.48 (6.46–10.58)	8.63 (6.60–10.65)
B	9.85 (3.62–18.41)	9.67 (4.10–16.77)
C	4.85 (1.20–8.77)	1.61 (0.04–4.05)
D	8.83(5.56–11.66)	8.55(5.97–11.83)
E	6.01 (1.02–11.77)	6.51 (0.07–12.65)

^a^Substitution rates are expressed as 10^−4^ substitutions per site per year. CR, Confidential range.

**Table 2:** Selection analysis of the S gene of OC43 genotypes.

Genotype	Mean dN/dS^a^	Site^b^	amino acid	dN/dS ± S.E.^c^	Pr^d^ (ω >1)	Subunit/Domain^e^
B	0.29	131	T	2.27 ± 1.50	0.658	NTD
		192	L	1.93 ± 1.58	0.536	NTD
		263	S	2.28 ± 1.50	0.663	NTD
		421	G	2.28 ± 1.50	0.648	RBD
		627	I	2.11 ± 1.55	0.603	S1
		951	Q	2.41 ± 1.51	0.710	S2
C	0.20	1001	T	2.43 ± 1.42	0.768	S2
D	0.31	33	N	6.15 ± 1.78	0.94	NTD
		38	P	5.56 ± 2.35	0.836	NTD
		90	K	5.94 ± 2.04	0.902	NTD
		93	T	5.90 ± 2.08	0.895	NTD
		115	T	3.72 ± 3.11	0.535	NTD
		120	D	6.17 ± 1.76	0.943	NTD
		176	Y	3.85 ± 3.11	0.556	NTD
		184	K	6.35 ± 1.50	0.976	NTD
		195	L	3.80 ± 3.11	0.548	NTD
		265	L	5.45 ± 2.42	0.818	NTD
		266	D	4.93 ± 2.66	0.731	NTD
		267	I	5.08 ± 2.60	0.755	NTD
		354	S	3.95 ± 3.10	0.572	RBD
		395	I	5.81 ± 2.16	0.878	RBD
		521	Y	6.47 ± 1.27	0.999	RBD
		535	F	5.32 ± 2.49	0.796	RBD
		716	Q	3.61 ± 3.11	0.518	S2
		741	Q	3.61 ± 3.11	0.517	S2
		763	R	6.47 ± 1.26	1.000	S2
		768	G	4.77 ± 2.78	0.706	S2
		782	V	6.38 ± 1.45	0.982	S2
		813	S	3.59 ± 3.11	0.515	S2
		989	L	5.67 ± 2.34	0.855	S2
		1255	D	4.97 ± 2.64	0.737	S2
		1310	C	3.87 ± 3.11	0.559	S2
E	0.15	NA	NA	NA	NA	

^a^dN/dS, the ratio of nonsynonymous (dN) to synonymous;

^b^The amino acid positions of genotype B were determined according to 87309 Belgium 2003 (AY903459) and 5240/07 (KF572844) for genotype C and D;

^c^S.E. Standard error;

^d^Pr, probability;

^e^NTD, N-terminal domain; RBD, receptor-binding domain; S, spike gene.
