# Supplementary material for: Genetic drift of human coronavirus OC43 spike gene during adaptive evolution
Source: Sci Rep. 2015 Jun 22;5:11451. doi: 10.1038/srep11451 (PMC4476415; doi:10.1038/srep11451)
Supplement: Supplementary Information [file srep11451-s1.pdf]

Genetic drift of human coronavirus OC43 spike gene during adaptive evolution

Lili Ren, Yue Zhang, Jianguo Li, Yan Xiao, Jing Zhang, Ying Wang, Lan Chen,

Gláucia Paranhos-Baccalà, Jianwei Wang

Figure S1. Amino acid variation of the S protein of OC43 genotype B strains from 2003 to 2012. The Belgium 2003 strain was used as reference sequence. The calculated positive selection sites are highlighted in grey. Positions predicted related to the chronological strains are highlighted in red.

Strain name	Amino acide position																	
	25	26	27	28	31	34	38	44	69	131	163	184	192	195	201	237	263	266
87309-BE03	T	S	L	K	F	R	S	D	L	T	L	K	L	L	S	F	S	D
HK04-04	-	-	R	T	·	·	P	A	I	·	·	·	·	M	·	V	·	·
HK04-07	-	-	R	T	·	·	P	A	I	·	·	·	·	M	·	V	·	·
HK04-08	-	-	R	T	·	·	P	A	·	·	·	·	·	M	·	V	·	·
HK04-10	-	-	R	T	·	·	P	A	I	·	·	·	·	M	·	V	·	·
HK04-17	-	-	R	T	·	·	P	A	I	·	·	·	·	M	·	V	·	·
1926/06	-	-	R	T	·	I	P	A	·	·	·	·	·	M	·	V	·	·
1908/10	-	-	R	T	·	I	P	A	·	·	·	·	S	M	P	V	·	·
1919/10	-	-	R	T	·	I	P	A	·	·	·	·	S	M	P	V	·	·
1997/10	-	-	R	T	·	I	P	A	·	·	V	N	·	M	·	V	A	N
2145/10	-	-	R	T	·	I	P	A	·	V	V	N	·	M	·	V	A	N
3184/12	-	-	R	T	L	I	P	A	·	·	·	N	·	M	·	V	·	N

Strain name	324	399	421	425	436	470	481	506	508	520	525	529	537	538	562	627	634	693	704	805	834	933	951
87309-BE03	N	C	G	Y	T	N	F	G	L	I	A	Y	N	P	C	I	Y	F	F	E	F	A	Q
HK04-04	·	·	·	·	·	·	·	·	·	·	·	·	·	·	·	·	·	·	·	·	Y	·	·
HK04-07	·	·	·	·	·	·	·	·	·	·	·	·	·	·	·	·	·	·	·	·	Y	·	·
HK04-08	·	·	·	·	·	·	L	S	·	·	·	H	·	·	R	K	H	·	·	·	Y	·	·
HK04-10	·	·	·	·	·	·	·	·	·	·	·	·	·	·	·	·	·	·	·	·	Y	·	·
HK04-17	·	·	·	·	·	·	·	·	·	·	·	·	·	·	·	·	·	·	·	·	Y	·	·
1926/06	·	G	·	·	S	·	L	S	S	M	·	·	·	·	·	K	·	·	·	·	Y	·	·
1908/10	H	·	F	·	·	·	L	S	·	·	·	·	·	·	·	K	·	·	·	·	Y	·	·
1919/10	·	·	·	·	·	·	L	S	·	·	V	·	·	·	·	K	·	·	·	·	Y	·	·
1997/10	·	·	·	·	·	·	L	S	·	·	·	·	T	L	·	K	·	Y	S	D	Y	S	·
2145/10	·	·	·	N	·	·	L	S	·	·	·	·	·	·	·	K	·	·	·	D	Y	·	S
3184/12	·	·	·	·	·	D	L	S	·	·	·	·	·	·	·	K	·	·	·	D	Y	·	·

Strain name	1006	1026	1030	1069	1160	1180	1236	1244	1264	1272	1275	1277	1289	1352	1355
87309-BE03	T	D	E	S	S	D	S	R	D	D	V	M	Q	Y	L
HK04-04	·	·	·	·	·	·	·	·	·	·	·	·	·	·	·
HK04-07	·	·	·	·	·	·	·	·	·	·	·	·	·	·	·
HK04-08	·	·	·	·	·	·	·	·	·	·	·	·	·	·	·
HK04-10	·	·	·	·	·	·	·	·	·	·	·	·	·	·	·
HK04-17	P	H	Q	A	N	N	·	K	·	·	·	·	H	·	·
1926/06	·	·	·	·	·	·	P	·	·	·	·	I	·	·	S
1908/10	·	·	·	·	·	·	·	·	·	·	·		·	·	·
1919/10	·	·	·	·	·	·	·	·	·	·	·	·	·	H	·
1997/10	·	·	·	·	·	·	·	·	G	·	·	·	·	·	·
2145/10	·	·	·	·	·	·	·	·	·	·	·	·	·	·	·
3184/12	·	·	·	·	·	·	·	·	·	N	F	·	·	·	·

NTD | RBD

S1 | S2

Figure S2. The alignment of NTD amino acids sequences of BCoV-Mebus strains with representative OC43 strains isolated in different years. The critical sugar-binding amino acids in BCoV, 171Y, 191E, 193W and 194H (numbering refer to 87309BE03) labeled with asterisk.

```
                  160       170       180       190       200       210       220
                 |....|....|....|*...|....|....|....|*.**|....|....|....|....|....
BCoV-Mebus   D--NKLQGLLEISVCQYTMCEYPHTICHPNLGNKRVELWHWDTGVVSCLYKRNFTYDVNADYLYFHFYQE
ATCC-VR759   .GY........V.....N.....Q.........H.K...L..............................
87309BE03    .GV........V.....N...........K...HFK...L...................T..........
HK04-02      .GV........V.....N...........K...HFK...L...................T..........
229/05       .GV........V.....N...........K...HFK...L...................T..........
HK06-01      .GV........V.....N...........K...HFK...L...................T..........
5240/07      .GV........V.....N...........K...HFK...L...................T..........
1034A/08     .GV........V.....N...........K...HFK...S...................T..........
1357A/09     .GV........V.....N...........K...HFK...S...................T..........
HK10-02      .GV........V.....N...........K...HFK...S...................T..........
HK11-01      .GV........V.....N...............HFK...S...................T..........
12689/12     .GV........V.....N...............HFK...L...................T..........
```

Table S1. Background information for the OC43 S gene sequences

Strain name	Accession No.	Sampling Year	Isolation sites	Genotype
87309 Belgium 2003	AY903459	2003	Belgium	B
19572 Belgium 2004	AY903460	2004	Belgium	D
HK04-01	JN129834	2004	Hong Kong, China	C
HK04-02	JN129835	2004	Hong Kong, China	D
HK04-03	NA [a]	2004	Hong Kong, China	C
HK04-04	NA	2004	Hong Kong, China	B
HK04-05	NA	2004	Hong Kong, China	C
HK04-06	NA	2004	Hong Kong, China	C
HK04-07	NA	2004	Hong Kong, China	B
HK04-08	NA	2004	Hong Kong, China	B
HK04-09	NA	2004	Hong Kong, China	C
HK04-10	NA	2004	Hong Kong, China	B
HK04-11	NA	2004	Hong Kong, China	C
HK04-12	NA	2004	Hong Kong, China	C
HK04-13	NA	2004	Hong Kong, China	C
HK04-14	NA	2004	Hong Kong, China	C
HK04-15	NA	2004	Hong Kong, China	C
HK04-16	NA	2004	Hong Kong, China	C
HK04-17	NA	2004	Hong Kong, China	B
HK04-18	NA	2004	Hong Kong, China	C
HK05-01	NA	2005	Hong Kong, China	C
HK05-02	NA	2005	Hong Kong, China	C
229/05	KF572816	2005	Beijing, China	C
1926/06	KF572807	2006	Beijing, China	B
3582/06	KF572817	2006	Beijing, China	C
3647/06	KF572818	2006	Beijing, China	C
HK06-01	NA	2006	Hong Kong, China	C
039A/07	KF572819	2007	Beijing, China	D
069A/07	KF572864	2007	Shandong, China	D
079A/07	KF572820	2007	Beijing, China	D
4795/07	KF572842	2007	Beijing, China	D
4954/07	KF572843	2007	Beijing, China	D
5240/07	KF572844	2007	Beijing, China	D
5331/07	KF572845	2007	Beijing, China	D
5345/07	KF572846	2007	Beijing, China	D
5352/07	KF572847	2007	Beijing, China	D
5370/07	KF572848	2007	Beijing, China	D
5414/07	KF572849	2007	Beijing, China	D
5442/07	KF572850	2007	Beijing, China	D
5445/07	KF572851	2007	Beijing, China	D
5472/07	KF572852	2007	Beijing, China	D
5479/07	KF572853	2007	Beijing, China	D
5484/07	KF572854	2007	Beijing, China	D
5485/07	KF572855	2007	Beijing, China	D
5508/07	KF572856	2007	Beijing, China	D
5517/07	KF572857	2007	Beijing, China	D
5519/07	KF572858	2007	Beijing, China	D
5566/07	KF572859	2007	Beijing, China	D
5595/07	KF572860	2007	Beijing, China	D
5617/07	KF572861	2007	Beijing, China	D

5625/07	KF572862	2007	Beijing, China	D
5656/07	KF572863	2007	Beijing, China	D
1034A/08	KF572824	2008	Beijing, China	D
1081A/08	KF572826	2008	Hebei, China	D
1135A/08	KF572827	2008	Hebei, China	D
1157A/08	KF572828	2008	Guangxi, China	D
1216A/08	KF572830	2008	Beijing, China	D
892A/08	KF572868	2008	Henan, China	D
978A/08	KF572872	2008	Shandong, China	D
HK08-01	NA	2008	Hong Kong, China	D
HK08-02	NA	2008	Hong Kong, China	D
HK09-01	NA	2009	Hong Kong, China	D
HK09-02	NA	2009	Hong Kong, China	D
8099/09	KF572865	2009	Beijing, China	D
8164/09	KF572866	2009	Beijing, China	D
8375/09	KF572867	2009	Beijing, China	D
9001/09	KF572870	2009	Beijing, China	D
9138/09	KF572871	2009	Beijing, China	D
1357A/09	KF572834	2009	Hebei, China	D
1382A/09	KF572835	2009	Henan, China	D
1591A/09	KF572836	2009	Sichuan, China	D
1593A/09	KF572837	2009	Hebei, China	D
10108/10	KF572821	2010	Beijing, China	D
10285/10	KF572822	2010	Beijing, China	D
10290/10	KF572823	2010	Beijing, China	D
10574/10	KF572825	2010	Beijing, China	D
1783A/10	KF572804	2010	Beijing, China	E
1908A/10	KF572805	2010	Beijing, China	B
1919A/10	KF572806	2010	Shanxi, China	B
1997A/10	KF572808	2010	Neimenggu, China	B
2058A/10	KF572809	2010	Beijing, China	E
2134A/10	KF572838	2010	Beijing, China	D
2145A/10	KF572810	2010	Shandong, China	B
2151A/10	KF572839	2010	Henan, China	D
HK10-01	NA	2010	Hong Kong, China	D
HK10-02	NA	2010	Hong Kong, China	D
HK11-01	NA	2011	Hong Kong, China	D
HK11-02	NA	2011	Hong Kong, China	D
2941A/11	KF572811	2011	Beijing, China	E
3074A/12	KF572812	2012	Hebei, China	E
3184A/12	KF572813	2012	Beijing, China	B
3194A/12	KF572814	2012	Henan, China	E
3269A/12	KF572841	2012	Beijing, China	D
12689/12	KF572831	2012	Beijing, China	D
12691/12	KF572832	2012	Beijing, China	D
12694/12	KF572833	2012	Beijing, China	D

[a]NA, not available, sequences were supported from the author published the paper (Lau *et al.*, 2011).

Lau, S.K. *et al.* Molecular epidemiology of human coronavirus OC43 reveals evolution of different genotypes over time and recent emergence of a novel genotype due to natural recombination. *J Virol* **85,** 11325-11337 (2011)
